# To FLASH or to Fractionate? That is the question

**DOI:** 10.1016/j.zemedi.2022.10.007

**Published:** 2022-10-31

**Authors:** Tony Lomax, Serena Psoroulas

**Affiliations:** Centre for Proton Therapy, Paul Scherrer Institute, Villigen, Switzerland

It cannot be disputed that the most high profile and discussed topic in radiotherapy today is FLASH. This is a biological effect whereby normal tissues appear to be better spared from radiation damage when irradiated at *dose rates* much higher than used conventionally (≫ 40 Gy/s). Although this effect has been known for many years, interest in the FLASH effect was recently renewed after publication of the seminal paper by Fauvadon et al. [1]. In addition to demonstrating substantial sparing of normal tissue reactions to high dose rate irradiations, the authors also demonstrated that there was *no* reduction in tumour response for the same high dose irradiations. Many papers have now been published confirming this effect for different pre-clinical models, and with different irradiation modalities such as electrons, protons or heavy ions [2], [3], [4], [5], [6], [7], [8], [9], [10]. Based on the (almost) exclusively positive results from pre-clinical studies, a first human patient was already treated using FLASH in 2019 at the University Hospital in Lausanne [11], [12], and some first clinical trials in cats (with electrons [13], [14]) and in humans (with high-energy protons [15] and electrons [16]) have been pursued.

But is FLASH the future of radiotherapy, or just another promising pre-clinical effect that ultimately fails to make it into the clinic? After all, in an editorial in the Journal of Translational Medicine in 2014, the authors reported that less than 1 in 10 of successful small animal pre-clinical studies in cancer research actually translate into human treatments [17].

## What are the conditions for triggering the FLASH effect?

As the name implies, FLASH can only be exploited if treatments are delivered at (very) high dose-rates. But what exactly is meant by dose-rate? The trivial answer is that it is dose divided by the time of irradiation. But here comes the ‘spoiler’. Over what time-scale is dose considered for this simple calculation? For instance, should dose-rate be based on the total time to deliver a full fraction or the time to deliver the dose of a single field? At the other end of the spectrum, as most delivery machines are pulsed at some level, should dose-rate not be calculated based just on the length of each pulse? And what about sequential irradiation techniques such as proton pencil beam scanning, where irradiation is not uniform in either space or time? All these definitions will of course give very different ‘dose-rates’. Unfortunately, in much of the FLASH literature, the exact definition of dose-rate, or more precisely, the time-scale over which dose-rate is determined, is not clearly defined. As such, even today, we still do not really know which dose-rate is important for the FLASH effect. Having said this, the community would likely agree that the total time to deliver the dose should be as short as possible and/or the in-pulse dose rate (if there is a strong pulse structure to the delivery) should be high [7]. The effects of pauses in delivery within a fraction (e.g., between the delivery of individual fields) or between fractions themselves are still, however, not well understood, even if pre-clinical studies are currently being performed to study these [5].

Whatever its definition however, dose-rate is likely not the only parameter that is important for triggering the FLASH effect. It is becoming increasingly clear that there may well be a *dose* dependence as well. That this could be the case has been recently demonstrated in the excellent review paper by Böhlen et al. [18]. In this, the authors reviewed much of the current FLASH literature, correlating the magnitude of the FLASH effect (i.e., the magnitude of normal tissue sparing) as a function of the delivered dose. Based on current knowledge, it thus appears that there may be little to no FLASH effect for (single shot) irradiations up to 10 Gy, after which the magnitude of the FLASH effect begins to slowly increase to about a 20% effect at 20 Gy and 30% at 40 Gy. It should of course be noted that there are inevitably large error bars on these figures and that observed effects will be dependent on the dose threshold for toxicity. Nevertheless, such a dose dependence could be important for understanding the future challenges for moving FLASH into the clinic.

## To FLASH or to fractionate? That is the question

This being an editorial for a medical physics journal, it may come as a surprise to hear that delivery technology is, we believe, the least of the issues of bringing FLASH into the clinic. Although many interesting developments still have to be made, in particular in the development of high dose-rate photon machines, modalities for the delivery of ultra-high dose rates (UHDR) to deep-seated tumours already exist. As demonstrated in the recently completed FAST01 trial [15], high dose rates can be safely delivered to bony metastases in extremities of patients using high energy protons (i.e., in so called ‘transmission mode’). For more superficial tumours, dedicated UHDR electron accelerators have been developed and it has been shown that conventional LINACS can be modified to deliver electrons at FLASH dose rates [18], [19] and are being used for a human trial [16]. In addition, methods for the delivery of very high energy electron treatments (VHEET) are currently under development [20]. As for photons, certainly much work still needs to be done to obtain FLASH dose-rates, although development of the PHASER, multi-LINAC system at Stanford, looks to be making promising progress [21]. On the other hand, although we know how to deliver ultra-high dose rates, not all UHDR deliveries necessarily trigger the FLASH effect [22]. As such, a detailed knowledge of the parameter space comprising dose (thresholds), total irradiation times, and beam characteristics in biological experiments where the FLASH effect is (or is not) observed is required before we can agree on a dose-rate definition that can allow us to design FLASH treatments which could be translated to the clinic.

A more far-reaching challenge for clinical FLASH is, we believe, the need to conform the dose to the tumour. Although it is tempting to believe that the normal tissue sparing characteristics of FLASH renders dose conformation to the tumour redundant, it is in fact well recognized in the FLASH community that dose conformation to the tumour is still necessary. This is clear when one considers that the maximum FLASH dose-modifying factors observed pre-clinically is about 0.6, but could be smaller for lower doses [18]. Even though such modifying factors would be very welcome and, if demonstrated in the clinical setting, could have a positive effect on reducing treatment related sequelae, they do not render doses delivered to normal tissue fully benign. Thus, dose conformation will still be an important element of any FLASH treatments.

This leads to what may be the biggest challenge facing the clinical implementation of FLASH. Dose conformation, whether with multiple fields, arcs and/or particles naturally aims to reduce the doses to normal tissues, the same tissues where we want the FLASH effect to be most effective. This leads to two important consequences for FLASH treatments.

First is what could be called the ‘conformation paradox’. The more we conform the dose, the lower the doses to normal tissues, and therefore the lower the likelihood (or magnitude) of the FLASH effect. Thus, an optimal balance will need to be found between the benefits of dose conformation and magnitude of the FLASH effectiveness, such that FLASH brings additional advantages to the patient over and above that already provided by dose conformation alone. This is made more complex if we assume that FLASH effectiveness could be organ and end-point dependent.

Second, given the likely dose levels required for FLASH, conventional fractionation regimes may have to be abandoned in order to exploit it in the clinic. This has important consequences. For instance, for a multiple field or ARC FLASH treatment delivered with photons, dose to the normal tissues may be about 50% of the dose delivered to the target. Thus, even if a (single fraction) dose of 40 Gy is delivered to the tumour, this implies that doses to large volumes of normal tissues would be about 20 Gy. According to the work of Böhlen et al., this may result in a ‘dose modifying factor’ of only about 0.8 (20%) [18]. But as fractionation must be abandoned, this dose modifying factor needs to be at least as effective in reducing normal tissue damage as the reductions of biological effectiveness dose are as a result of fractionation. Whether this is the case is a (very important) open question. Böhlen et al. recently published a break-even analysis on the topic [23], but the current biological knowledge limits the validity of predictions, particularly when looking at strongly hypofractionated schedules. Results from a recently abandoned FLASH clinical trial treating cats would indicate that, at least for some late treatment related sequelae (bone necrosis), such a break-even point is yet to be reached [14].

## Is there a future for FLASH?

It is probably too early to say what the future of FLASH is clinically. Nevertheless, much important and very interesting work is being pursued, and further clinical trials in patients are being planned, mostly of the phase 1 type to show that high dose rate treatments can be delivered safely, rather than with the aim to demonstrate the FLASH effect clinically. But assuming that such trials are successful, what are the likely advantages that FLASH will bring?

The most obvious is a hoped for reduction of treatment related sequelae, although such reductions, predominantly shown for acute reactions in the pre-clinical setting, also need to be shown for late toxicities. In addition, there are some hints that high dose rate treatments could be more effective for hypoxic tumours. Although it is early days yet, and these results need to be confirmed and understood, this is certainly an exciting prospect.

On the other hand, maybe there is no need to show that FLASH is advantageous to conventional, fractionated treatments - as long as it can be shown to be at least equally effective.

For instance, although fractionated radiotherapy is considered a ‘non-invasive’ treatment, it is rather invasive on a patient’s time. If the normal tissue sparing effects of fractionation can thus be ‘substituted’ by the FLASH effect, even if only in a sub-set of patients and indications, it could have a huge impact on the quality of life for patients during treatment, drastically reducing the number of hospital visits required to complete their treatment. In addition, the development of UHDR delivery systems, developments driven by the current FLASH fad, will likely lead to more time efficient treatments (reduced delivery times) and the potential for significantly increased patient throughputs. As such, it could be that one of the main, and more immediate benefits of the current FLASH craze could actually be reduced treatment costs and a consequent increase in the cost effectiveness of radiotherapy.

But we believe, there is an even more fundamental advantage that could result from the current interest in FLASH. The radio-biology debate on FLASH at this year's ESTRO conference had been one of the most attended sessions of the whole conference. As such, there is a renewed interest in radiobiology and radiochemistry which could potentially last beyond the current hype, bringing these disciplines back into the spotlight.
